# Risk and Preventive Factors for SUDI: Need We Adjust the Current Prevention Advice in a Low-Incidence Country

**DOI:** 10.3389/fped.2021.758048

**Published:** 2021-11-16

**Authors:** Floortje Kanits, Monique P. L'Hoir, Magda M. Boere-Boonekamp, Adèle C. Engelberts, Edith J. M. Feskens

**Affiliations:** ^1^Department of Human Nutrition and Health, Wageningen University, Wageningen, Netherlands; ^2^Community Health Center, GGD Noord-Oost-Gelderland, Warnsveld, Netherlands; ^3^Department of Health Technology & Services Research, University of Twente, Enschede, Netherlands; ^4^Department of Pediatrics, Zuyderland Medical Center, Sittard-Geleen, Netherlands

**Keywords:** SIDS (sudden infant death syndrome), SUDI (sudden unexpected death in infancy), incidence, risk factors, preventive factors, high-risk groups, prevention advice

## Abstract

**Background:** The incidence of Sudden Unexpected Death in Infancy (SUDI) is low in the Netherlands, with an incidence rate of 0.18 per 1,000 live births. Therefore, prevention advice may receive less attention, potentially leading to increasing incidence rates. It is currently unknown whether the risks for SUDI changed in the Netherlands, and if other risk factors might be present. The aim of this study was to examine the current risks and preventive factors for SUDI in Dutch infants, in order to determine if it is necessary to adapt the prevention advice toward the current needs.

**Methods:** A case-control study was conducted comparing SUDI cases aged <12 months from 2014–2020 in the Netherlands (*n* = 47), to a Dutch national survey control group from 2017 including infants <12 months of age (*n* = 1,192).

**Results:** Elevated risks for several well-known factors were observed, namely: duvet use (aOR = 8.6), mother smoked during pregnancy (aOR = 9.7), or after pregnancy (aOR = 5.4) and the prone sleeping position (aOR = 4.6). Reduced risks were observed for the well-known factors: room-sharing (aOR = 0.3), sleep sack use (aOR = 0.3), breastfeeding (aOR = 0.3), and the use of a pacifier (aOR = 0.4). For infants <4 months, the risk for SUDI was higher when bed-sharing (aOR = 3.3), and lower when room-sharing (aOR = 0.2) compared to older infants. For older infants, the sleep sack was found to be more protective (aOR = 0.2). A high risk for SUDI when bed-sharing was found when mother smoked, smoked during pregnancy, or if the infant did not receive any breastfeeding (respectively aOR = 17.7, aOR = 10.8, aOR = 9.2).

**Conclusions:** Internationally known factors related to the sudden unexpected death of infants were also found in this study. Relatively new findings are related to specific groups of infants, in which the strengths of these risk factors differed. In a low-incidence country like the Netherlands, renewed attention to the current prevention advice is needed. Furthermore, additional attention for prevention measures in low educated groups, and additional advice specifically targeting high-risk groups is recommended.

## Introduction

Sudden Unexpected Death in Infancy (SUDI) is a broad term used to describe the sudden unexpected death of an infant without an apparent cause, which includes Sudden Infant Death Syndrome (SIDS). SIDS is “the sudden unexpected death of an apparently healthy infant under one year of age that remains unexplained after a thorough case investigation, including performance of a complete autopsy with ancillary testing, examination of the death scene, and review of the clinical history,” and often occurs during an unobserved sleep period (1, 2). Taylor et al. (3), proposed a set of six codes from the International Classification of Diseases-10 (ICD-10), to encompass the majority of SUDI in eight high-income countries, to ensure better international comparison. This set includes SIDS (R95), and for example accidental suffocation or strangulation in bed (W75) and other ill-defined and unspecified causes (R99).

Both the incidence of SIDS and SUDI have largely declined in high-income countries since the 1980s, when the advice was given not to place infants to sleep prone (4). Between 2002 and 2010, low incidence rates were observed in the Netherlands (0.19 per 1,000 live born infants) (3). Nevertheless, 31 infants died suddenly and unexpectedly in 2019, of which 13 were classified as SIDS (5). The loss of a child is considered among the worst experiences in life, and the traumatic aspect of the unexpected death leads to great parental grief and psychological burden (6). Parents experience intense feelings of responsibility and failure in their role as parents, and most of these difficulties are under-recognized and unaddressed.

Various risk factors, as well as preventive factors for SUDI have been recognized internationally over the years (7). These findings have been incorporated in specific prevention programs, which have contributed greatly to the decrease in incidence. Risk factors can be divided in unavoidable and avoidable factors. Unavoidable risk factors are the infant's young age, male gender, being second or later born, and born small for gestational age and/or prematurely. Potentially avoidable risk factors include the prone and side sleeping position, unsafe bedding, parental smoking, and unsafe sleeping places, including bed-sharing. Preventive factors include breastfeeding, appropriate sleep sack use, consistent pacifier use, and room-sharing in a separate bed. These factors are incorporated in the Dutch guideline for the prevention of SUDI (8). The most recently updated prevention campaign in The Netherlands is that of The Dutch Consumer Safety Institute (Veiligheid NL), which focuses on “The four of Safe Sleeping”: sleeping supine, in an own cot or crib, in a well-fitting sleep sack, and in an empty bed without soft materials (9).

SUDI prevention advice in the Netherlands is successively offered by the midwife, the maternity nurse and the preventive child healthcare physician and nurse, and is characterized by a continuous supply of information and care. The organization and approaches of youth healthcare (YHC) differ between countries (10). In the Netherlands, the YHC includes free governmentally established preventive care for all children 0–18 years of age, provided by professionals who monitor growth and development of children, and carry out the vaccination program. With regard to the first year of life, infants and their parent(s) have 6–8 consultations with the preventive child healthcare center (PCHC). PCHC attendance during the first year of life is high in the Netherlands. This preventive system, together with obstetric and maternity care, offers many opportunities to interact with parents about infant care practices.

Since the incidence of SUDI is low in the Netherlands, attention for prevention can fade among parents and (professional) caregivers, as well as in governmental organizations and (public) health professionals, potentially leading to increasing incidence rates. Furthermore, parental behavior changes over time, and new trends regarding infant care arise. In 2007, the original 1996 consensus statement on SIDS prevention was rewritten into a prevention guideline. This guideline was revised in 2009 and a multidisciplinary national cooperation agreement was written in 2017 to aid implementation. The main messages remained unchanged. Studies are needed to determine if it is necessary to adapt the prevention advice toward the current needs. Therefore, this study aimed to identify risk and preventive factors for SUDI and their prevalence in Dutch infants under 12 months of age in the period 2014–2020, and to explore these factors in high-risk groups.

## Materials and Methods

A case-control study was conducted comparing SUDI cases aged 0–12 months from 2014 to 2020 in the Netherlands, to a national survey control group from 2017.

### Data Collection

The case group consisted of SUDI cases aged 0–12 months who died in the period 2014 up to 2020 in the Netherlands, and were reported to the SUDI Expert Group of the Dutch Pediatric Society. The Expert Group consists of a group of pediatricians, pathologists, a pediatric cardiologist, pediatric physiotherapist, youth health doctor/epidemiologist, biologist, and a psychotherapist, who review the reported SUDI cases. Classification of these cases is based on the Avon clinico-pathological system (11). Upon consent of the parents, the pediatricians visit the families of reported SUDI cases a few weeks after their loss, and fill in an extensive questionnaire together with the parents. The Expert Group has registered these data in a database since 1996.

Data of an unmatched control group were retrieved from a Dutch national survey on safe sleeping conditions in 2017 (12). For this survey, parents with an infant under 12 months old were asked to fill in an online survey via a link on a flier that was distributed among 139 PCHCs. To also include a representative number of respondents with lower socioeconomic status (SES), 21 PCHCs in areas of low SES were selected to also conduct paper questionnaires. Furthermore, via online media, parents were asked to fill in the online survey.

Previous approval process allow for the use of this de-identified data from both cases and controls.

### Data Assessment

For all cases and controls, the mother's migration background was defined by the country she was born in, and categorized as either Dutch or non-Dutch. The mother's education level, as indicator of SES, was defined by the highest educational level attained, categorized as low, middle, or high, based on the division used in the Netherlands (13).

Unavoidable risk factors for SUDI, including infant age in months, gender, birth rank and birthweight, were assessed among cases and controls. Birth rank indicates either the first, second, or third or later born infant of the mother. Birthweight was dichotomized into under 2,500 grams (low), or 2,500 grams or higher (normal). Avoidable risk factors and preventive factors for SUDI that were assessed included: sleeping position, bed-sharing, room-sharing, duvet use, sleep sack use (wearable blanket), pacifier use, breastfeeding and maternal smoking both during and after pregnancy.

Sleeping position, i.e., placed to sleep in the prone, side or supine position, was assessed for last night (controls), or last time before death (SUDI cases). Bed-sharing was defined as sleeping with one or both parents in bed for most of the last night (controls), and sharing the sleep-surface with one or both parents when the infant was found dead (SUDI cases). The type of bedding, including a duvet and sleep sack, the infant was covered with last night was assessed for controls, and for cases it was the type of bedding when found deceased. Room-sharing for controls was assessed by the sleeping place where the infant slept most of last night, including sleeping in the parents' bedroom in an own crib or cot, or in a co-sleeper. For the cases this was assessed by the infant sleeping in the parents' bedroom, but not bed-sharing, during the last sleep. Pacifier use was assessed regarding the usual way the infant was placed to sleep for both cases and controls. Infant feeding type and smoking of the mother were assessed at the time of filling out the questionnaire for controls, whereas for the SUDI cases this was around time of death. Breastfeeding included both exclusive breastfeeding, and breastfeeding supplemented with formula feeding. Lastly, maternal smoking during pregnancy was asked for both cases and controls.

### Data-Analysis

As the control population comprised a relatively high number of highly educated parents, data of the 2017 survey were weighted according to the education level distribution of women aged 25–45 in 2017, as retrieved from the Central Bureau of Statistics Netherlands (14). This resulted in the following weighting factors: 0.936 for low education level; 1.292 for medium education level; and 0.867 for high education level.

Firstly, background characteristics of SUDI cases and their weighted survey controls were generated. Secondly, prevalence of risk and preventive factors among both cases and controls were presented. With Logistic Regression analyses, Odds Ratios (ORs) with 95% Confidence Intervals (CI) were calculated. Adjusted ORs (aORs) were calculated to correct for potential confounding of non-modifiable factors related to SUDI risk (age, gender, birthweight and birth rank). Data on aORs, combined with the prevalence of the risk factor or inverse prevalence of the preventive factor among SUDI cases, were used to calculate the Population Attributable Fraction (PAF) with the formula: $PAF=\frac{\left(OR-1\right)}{OR}\;\times prevalence$. By explaining the theoretical percentage of SUDI cases in the total population that could be attributed to the specific risk or preventive factor, the PAF provides an estimation of the relative impact on SUDI incidence that could be achieved if the risk were reduced or eliminated, while all other factors remained constant. Specific groups of infants are known to be at higher risk of SUDI when exposed to certain risk factors, and the current prevention advice might need specification for these groups. Therefore, stratified risks (ORs and aORs with 95% CIs) were calculated for infants <4 months and ≥4 months, and for infants with low and normal/high birthweight. Furthermore, the risk of bed-sharing was separately explored over various strata, as it is still unclear whether specific groups are at higher risk of SUDI when bed-sharing compared to others.

## Results

Between 2014 and 2020, 56 SUDI cases were reported to the Expert Group. Nine cases were excluded because of: missing date of birth (13); over 12 months of age (4); a cause of death was subsequently found (15). Therefore, 47 SUDI cases were included in this study.

Parents of 1,209 infants participated in the national survey on safe sleeping in 2017. After weighing for education level, 1,192 controls could be used for analyses. Characteristics of the 47 cases and 1,192 weighted controls are presented in Table 1. Mean age of controls was slightly older than that of cases. Furthermore, cases were more often boys, second or later born and born with low birthweight. Mothers of cases were more often non-Dutch, and lower educated compared to the mothers of controls.

**Table 1:** Characteristics of cases and controls.

		**Cases**		**Controls**	
		***n*** **=** **47**		***n*** **=** **1,192**	
Age^∧^		3.4 ± 2.7		5.1 ± 3.4	
Gender	Boy	28	(59.6%)	586	(49.2%)
	Girl	19	(40.4%)	605	(50.8%)
Birth rank	First	14	(32.5%)	658	(55.2%)
	Second	21	(47.5%)	375	(31.5%)
	Third or more	9	(20.0%)	159	(13.3%)
Birthweight	≥2,500 g	38	(80.9%)	1,143	(95.9%)
	<2,500 g	9	(19.1%)	49	(4.1%)
Migration background mother	Dutch	34	(77.3%)	1,032	(87.0%)
	Non-Dutch	10	(22.7%)	154	(13.0%)
Education level mother	Low	6	(15.0%)	157	(13.2%)
	Middle	19	(47.5%)	443	(37.2%)
	High	15	(37.5%)	591	(49.6%)

*Frequency and percentage of population with non-missing data for respective factors are presented. Controls are weighed for maternal education level*.

∧ *Age is presented as mean ± standard deviation*.

Table 2 shows ORs and PAFs for risk and preventive factors for SUDI. All studied risk factors were found to have a higher prevalence among SUDI cases compared to controls in univariate analyses, except for the factor placed in the side sleeping position. No significantly elevated risk was found for bed-sharing in the total group. The risk of SUDI for an infant placed to sleep in the prone position was 4.6 (2.1–10.3) times as high as the risk for infants placed supine. Data suggest that around 21% (PAF% = 21.4) of the SUDI cases could possibly have been prevented if these infants had been placed supine. As over one third of mothers of SUDI cases smoked either during or after pregnancy, and aORs were respectively 9.7 (4.6–20.4) and 5.4 (2.6–11.4), high PAFs of respectively 31.3 and 28.4% were found.

**Table 2:** Prevalence in cases and controls of known risk and preventive factors for SUDI, Odds Ratio (OR), both crude and adjusted, and the Population Attributable Fraction (PAF) for the aOR.

	**Cases *n* = 47**	**Controls *n* = 1,192**	**OR**	Adjusted OR^*^	**PAF (%)**
**Risk factors**					
Position placed to sleep*prone vs supine*	12 (27.3%)	105 (8.9%)	3.9 (1.9–7.8)	4.6 (2.1–10.3)	21.4
*side vs supine*	3 (6.8%)	99 (8.4%)	1.0 (0.3–3.4)	1.0 (0.3–3.4)	-
Bed-sharing*yes vs no*	7 (16.3%)	118 (10.0%)	1.8 (0.8–4.0)	2.0 (0.8–4.7)	8.2
Duvet*yes vs no*	10 (24.4%)	55 (4.6%)	6.6 (3.1–14.2)	8.6 (3.7–20.2)	21.6
Mother smoked during pregnancy*yes vs no*	15 (34.9%)	47 (3.9%)	13.1 (6.5–26.1)	9.7 (4.6–20.4)	31.3
Mother smokes after pregnancy*yes vs no*	15 (34.9%)	78 (6.6%)	7.6 (3.9–14.8)	5.4 (2.6–11.4)	28.4
**Preventive factors**					
Sleep sack*yes vs no*	9 (24.3%)	657 (55.1%)	0.3 (0.1-0.6)	0.3 (0.1-0.7)	52.0
Breastfeeding*exclusive/mixed vs none*	12 (26.7%)	494 (41.6%)	0.5 (0.3-1.0)	0.3 (0.2-0.7)	48.9
Room-sharing, not bed*yes vs no*	10 (21.7%)	362 (30.6%)	0.6 (0.3–1.3)	0.3 (0.1–0.6)	57.1
Usually pacifier*yes vs no*	17 (43.6%)	699 (58.7%)	0.5 (0.3–1.0)	0.4 (0.2–0.8)	33.8

* *Adjusted for infant age, gender, birthweight and birth rank. Due to adjustment, a maximum of two extra missing cases was present per factor*.

All studied potential preventive factors were found to have lower prevalence among SUDI cases compared to controls. Combining ORs with the prevalence of not performing the preventive behavior resulted in varying PAFs. The highest PAF was found for room-sharing, where, if all infants had slept in the parents' bedroom, but not in the parental bed, potentially 57% of cases could have been prevented [aOR room-sharing 0.3 (0.1–0.6)]. The lowest PAF was found for infants usually not placed to sleep with a pacifier, where the PAF was almost 34%. Infants usually provided with a pacifier when placed to sleep had 0.4 (0.2–0.8) times the risk of SUDI compared to those without. Infants sleeping in a sleep sack or receiving any breastfeeding had 0.3 times the risk of SUDI (95% CI 0.1–0.7 and 0.2–0.7 respectively) compared to infants who did not.

To explore risk factors (placed prone, bed-sharing, duvet) and preventive factors (sleep sack, room-sharing, pacifier) in specific groups of infants for which the current prevention advice may require specification, stratified analyses were performed and results are summarized in Table 3. For infants under the age of 4 months, the risk of SUDI when placed in the prone position was 6.9 (2.0–23.9) times as high as when placed supine, and 12.6 (0.9–168.3) times as high for infants with a birthweight under 2,500 grams. While there was no strong evidence for an increased risk of SUDI when bed-sharing for the total group, a high risk was found for infants under the age of 4 months [aOR = 3.3 (1.1–9.3)], for infants who did not receive any breastfeeding [aOR = 9.2 (3.0–28.6)], and for infants whose mother smoked after pregnancy [aOR = 17.7 (1.9–162.8)] or during pregnancy [aOR = 10.8 (1.4–81.3)] (Supplementary Table 1). Furthermore, a high risk for duvet use was observed in both the younger infants, and those with low birthweight. The preventive effect of a sleep sack was found to be greater among infants 4 months of age and older [aOR = 0.2 (0.1–0.6)], compared to infants aged under 4 months [aOR = 0.5 (0.1–2.0)]. These infants under the age of 4 months also had 0.2 (0.0–0.5) times the risk of SUDI when room-sharing, which was lower than 0.6 (0.2–2.2) times the risk for the older infants.

**Table 3:** Prevalence in cases and controls of known risk and preventive factors for SUDI, stratified for age and birthweight, and the Odds Ratio (OR), both crude and adjusted in these strata.

	**Cases**	**Controls**	**OR**	Adjusted OR^*^
**Risk factors**				
Position placed to sleep*prone vs supine*	12 (27.3%)	105 (8.9%)	3.9 (1.9–7.8)	4.6 (2.1–10.3)
*Age <4 mo*	5 (21.7%)	23 (4.8%)	6.0 (2.0–17.9)	6.9 (2.0–23.9)
*Age ≥4 mo*	7 (33.3%)	82 (11.6%)	3.4 (1.4–8.8)	3.5 (1.2–10.3)
*Birthweight <2,500 gr*	2 (22.2%)	3 (6.3%)	4.1 (0.6–28.3)	12.6 (0.9–168.3)
*Birthweight ≥2,500 gr*	10 (28.6%)	101 (8.9%)	4.0 (1.9–8.7)	4.3 (1.8–10.1)
Bed-sharing*yes vs no*	7 (16.3%)	118 (10.0%)	1.8 (0.8–4.0)	2.0 (0.8–4.7)
*Age <4 mo*	6 (26.1%)	44 (9.1%)	3.5 (1.3–9.4)	3.3 (1.1–9.3)
*Age ≥4 mo*	1 (5.0%)	75 (10.6%)	0.4 (0.1–3.4)	0.5 (0.1–4.2)
*Birthweight <2,500 gr*	1 (11.1%)	3 (6.2%)	1.9 (0.2–20.4)	1.4 (0.1–21.9)
*Birthweight ≥2,500 gr*	6 (17.6%)	115 (10.2%)	1.9 (0.8–4.7)	2.0 (0.8–5.0)
Duvet*yes vs no*	10 (24.4%)	55 (4.6%)	6.6 (3.1–14.2)	8.6 (3.7–20.2)
*Age <4 mo*	7 (30.4%)	12 (2.5%)	16.6 (5.8–47.5)	17.6 (5.3–57.8)
*Age ≥4 mo*	3 (16.7%)	43 (6.1%)	3.1 (0.9–11.2)	5.6 (1.4–22.4)
*Birthweight <2,500 gr*	3 (42.9%)	2 (4.1%)	16.2 (2.1–122.6)	386.5 (2.0–74,013.4)
*Birthweight ≥2,500 gr*	7 (20.6%)	53 (4.6%)	5.3 (2.2–12.8)	7.1 (2.8–18.1)
**Preventive factors**				
Sleep sack*yes vs no*	9 (24.3%)	657 (55.1%)	0.3 (0.1–0.6)	0.3 (0.1–0.7)
*Age <4 mo*	3 (15.8%)	141 (29.2%)	0.5 (0.1–1.6)	0.5 (0.1–2.0)
*Age ≥4 mo*	6 (33.3%)	516 (72.7%)	0.2 (0.1–0.5)	0.2 (0.1–0.6)
*Birthweight <2,500 gr*	0 (0.0%)	26 (54.2%)	-	-
*Birthweight ≥2,500 gr*	9 (29.0%)	631 (55.2%)	0.3 (0.2–0.7)	0.4 (0.2–1.0)
Room-sharing, not bed*yes vs no*	10 (21.7%)	362 (30.6%)	0.6 (0.3–1.3)	0.3 (0.1–0.6)
*Age <4 mo*	6 (25.0%)	246 (51.5%)	0.3 (0.1–0.8)	0.2 (0.0–0.5)
*Age ≥4 mo*	4 (18.2%)	116 (16.5%)	1.1 (0.4–3.4)	0.6 (0.2–2.2)
*Birthweight <2,500 gr*	5 (55.6%)	14 (29.0%)	3.1 (0.7–13.1)	1.3 (0.2–7.6)
*Birthweight ≥2,500 gr*	5 (13.5%)	348 (30.7%)	0.4 (0.1–0.9)	0.2 (0.1–0.5)
Usually pacifier*yes vs no*	17 (43.6%)	699 (58.7%)	0.5 (0.3–1.0)	0.4 (0.2–0.8)
*Age <4 mo*	9 (45.0%)	303 (62.9%)	0.5 (0.2–1.2)	0.4 (0.1–1.0)
*Age ≥4 mo*	8 (42.1%)	396 (55.9%)	0.6 (0.2–1.4)	0.4 (0.1–1.0)
*Birthweight <2,500 gr*	3 (50.0%)	31 (63.3%)	0.6 (0.0.1–3.2)	0.4 (0.0–2.8)
*Birthweight ≥2,500 gr*	14 (42.4%)	669 (58.5%)	0.5 (0.3–1.1)	0.4 (0.2–0.8)

* *Adjusted for infant age, gender, birthweight and birth rank. N cases: total = 47, <4 mo = 25 (53.2%), ≥4 mo = 22 (46.8%), <2,500 gr = 9 (19.1%), ≥2,500 gr = 38 (80.9%) N controls: total = 1,192, <4 mo = 482 (40.5%), ≥4 mo = 710 (59.5%), <2,500 gr = 49 (4.1%), ≥2,500 gr = 1,143 (95.9%)*.

## Discussion

In the current study, risk and preventive factors for SUDI and their prevalence in Dutch infants under 12 months of age were identified for the period 2014–2020. Significantly elevated risks were found for infants placed under a duvet, infants whose mother smoked pre- and/or postnatally, and infants placed in the prone sleeping position. Significantly reduced risks were found for room-sharing, sleeping in a sleep sack, breastfeeding, and the usual use of a pacifier. These are internationally known factors related to the sudden unexpected death of infants (7, 16–20).

Relatively new is that the strengths of these risk factors differed among specific groups of infants. A high risk of SUDI was found for infants under 4 months of age when placed prone, bed-sharing or placed under a duvet. In these young infants, room-sharing with parent(s) greatly reduced the risk. Infants aged 4 months and older benefit most from the preventive effect of a sleep sack. For infants born with low birthweight (under 2,500 grams), sleeping in the supine position is particularly important as they are at higher risk for SUDI when placed prone. For the total group of infants, there was no strong evidence for an increased risk of SUDI when bed-sharing. However, a significantly high risk was found for young infants, infants whose mother currently smokes, or smoked during pregnancy, and infants not receiving any breastfeeding.

Besides ongoing attention for the current prevention advice, additional focus should be on risk factors with the most impact on the population risk of SUDI, assessed by the PAF which is a combination of the OR and the prevalence of the risk factor. According to the results of this study, several SUDI cases could possibly have been prevented by room-sharing of parent(s) and infant, and by placing the infant to sleep in a sleep sack. Therefore, additional attention is necessary regarding room-sharing, especially with infants under 4 months of age, and the use of a sleep sack, especially for infants over 4 months of age.

Compared to an earlier Dutch study in the period 1996–2001 (21), the magnitude of risk increasing and preventive factors in the current study varies, but with overlapping confidence intervals. In terms of preventive factors, in the current study a stronger preventive effect was seen for sleeping in a sleep sack (aOR 0.3 vs. 0.7), for breastfeeding (aOR 0.3 vs. 0.5), and for pacifier use (aOR 0.6 vs. 1.0), compared to the earlier study. Data on room-sharing were not reported by De Jonge et al. (21). In the Netherlands, the use of sleep sacks and pacifiers has increased over the past decades, as can be observed from the data of the control populations (21). In terms of risk factors, now a higher risk of SUDI was found compared to the earlier study for sleeping prone (aOR 4.6 vs. 3.0), placed under a duvet (aOR 8.6 vs. 3.9), and smoking after pregnancy (aOR 5.4 vs. 2.7). The estimated risks for bed-sharing were comparable. No notable differences in the prevalence of prone sleeping and bed-sharing between the control populations of both studies were observed, but the use of a duvet and smoking of parents were much lower in 2017 compared to the earlier study (21).

The association between bed-sharing and SUDI is subject of international debate. Although there was no strong evidence of an increased risk of SUDI for the total group in the current study when bed-sharing, the risk was estimated to be extremely high in different sub-groups, and therefore still of major concern. The prevalence of bed-sharing in the parents' bed increased in the period 2002-2017 in the Netherlands (12, 22). This is especially of concern for infants under 4 months, where the associated risk of SUDI is higher, and the prevalence of bed-sharing was 9.1% in 2017. The risk of bed-sharing was also higher for infants whose mother smoked. Similar results were found in a case-control study combining individual data from a European, Scottish, New Zealand, Irish and German database (23). The same study also showed an increased risk of SUDI when bed-sharing with a parent who used alcohol or drugs (23). As this information was lacking in the current study, we weren't able to confirm this. Furthermore, the risk of bed-sharing in the current study was increased among formula fed infants. There was no significant risk associated with bed-sharing among breastfed infant, but numbers are very small. In the Netherlands, breastfeeding prevalence is higher among high educated mothers (90% at birth, and 51% at 5 months after birth) compared to low educated parents (69% at birth, 33% at 5 months after birth) (24). Also, smoking is more prevalent among low educated people (age 25–44: men 55%, women 40%) compared to high educated people (age 25–44: men 17%, women 13%) (15). This indicates a cumulation of risk factors (no breastfeeding and smoking) in lower educated parents, and thereby an increased risk when bed-sharing, making this an important target group for specific SUDI prevention strategies.

Most factors found in this study point in the direction of accidental suffocation, or accidental asphyxia contributing to the sudden death of an infant. Physiological studies indicate that facial obstruction in infants by e.g., soft bedding or lying face straight down, may lead to complete upper airway obstruction and/or accidental suffocation by rebreathing, and/or overheating (25). In these cases, it might be assumed that the airway-protective components of the infant's arousal response failed (26). It is known that maternal smoking impairs infant arousal processes (27), and higher arousal thresholds are also found among preterm born infants, infants sleeping prone, infants that are too warm/overheated and among formula-fed infants (28). Some SUDI cases while bed-sharing might be designated as accidental suffocation in bed (29). For infants under 4 months of age, this can be the case as they lack motor skills to escape potential threats in the parents' bed, when for example being covered with soft bedding (29). Well-developed upper airway muscle tone might contribute to the prevention of airway obstruction in hazardous situations, which might be stimulated by breastfeeding and pacifier sucking (30–32). Furthermore, overlaying when sharing a surface can obstruct the airways either directly, or by inadvertent pressure on the infant's lower jaw (33).

The prone sleeping position is a well-known risk factor for SUDI. Studies also identified a similar risk of the side sleep position, likely because many infants who are placed on their side can roll to the prone position, or lay with their face against (soft) bed material. Nevertheless, no elevated risk for infants being placed in the side position compared to the supine position was found in the current study. Infants with a birthweight under 2,500 gram, i.e., those born small for gestational age and/or prematurely are at higher risk when sleeping prone. The AAP guideline advises to “keep hospitalized preterm infants predominantly in the supine position, at least from 32 weeks gestational age onwards, so that they become acclimated to supine sleeping before discharge” (16). It would be insightful to gather data if this guideline is adhered to in the Netherlands. If not, it offers opportunity for extra targeted prevention through neonatal and newborn units.

Both the use of a pacifier, and the use of a sleep sack can reduce the risk of SUDI, which may be reflected by the same mechanism of preventing infants from turning to the prone position in their cot or crib. Around the age of 5 months, most infants start rolling from the supine to the prone position, but may still have problems rolling back. This can explain the lower risk of SUDI among infants over 4 months old placed in a sleep sack, compared to younger infants: a sleep sack can delay turning over to the prone position as it hampers the infant slightly, especially in using their legs as a fulcrum to turn (34). Consistent use of a pacifier may soothe the infant and support falling asleep, whereby turning prone might be inhibited. It is also suggested that pacifier sucking improves airway stabilization, and thereby contribute to the prevention of SUDI (35). It should be noted that it is important that the pacifier is used consistently with every sleep (36).

### Strengths and Limitations

A strength of this study is that we were able to assess multiple risk and preventive factors for SUDI among both cases and controls. The Dutch practice of having SUDI cases and their families visited at home by an Expert group pediatrician who interviews the parents and conducts an extensive questionnaire, has resulted in detailed information of the included cases. The 2017 survey used for the control population included comparable questions, which allowed for a good comparison between the groups. The control population was a good representative of the general Dutch population, however, slightly more first born infants and less infants with a non-Dutch mother were included (12).

A limitation of this study is that the size of the case group is rather small. This is mainly due to the low incidence of SUDI in the Netherlands. Furthermore, SUDI cases are only reported to the SUDI expert group when parents give their consent to a visit of one of the members. As not all parents provide consent, not all SUDI cases in the Netherlands are reported to the SUDI Expert group. Underreporting seems especially to be the case for non-Dutch parents who have lost their child, what may have led to an underrepresentation of this group among the cases. The small size of the case group means that, when exploring subgroups of infants and risk factors for SUDI among these groups, there might not be enough power to show statistically significant results.

## Conclusion

While the risk of SUDI in the Netherlands is still low, the current study shows several factors that significantly increase this risk. Therefore, focus on “the four of safe sleeping” factors in the current primary prevention should be maintained. This implies that renewed attention by midwives, maternity nurses and preventive child healthcare physicians and nurses is needed for these infant care factors. Besides, a cumulation of risk factors in low educated parents can be observed, indicating a need for additional attention for prevention measures in this group. A new selective prevention campaign regarding bed-sharing should be initiated in the PCHCs, focusing on parents of infants under 4 months of age, parents who smoke, and those who formula feed their infant. The use modern, picture driven prevention information material is recommended to reach as many groups in society as possible.

## Data Availability Statement

The data analyzed in this study is subject to the following licenses/restrictions: Granted permission is required to access datasets. Requests to access these datasets should be directed to https://easy.dans.knaw.nl/ui/datasets/id/easy-dataset:110196 for survey datasets, and requests for SUDI cases data should be submitted to the NODOK science committee, which can be arranged *via* the fourth author: a.engelberts@zuyderland.nl.

## Author Contributions

All authors made substantial contributions to the conception, design, and interpretations of the work. FK performed the analyses of the data. All authors assisted in preparing the article, critically assessed the final version, and agree to be accountable for the accuracy and integrity of the work.

## Funding

This research is funded by the Wageningen University, Department of Human Nutrition and Health and Department of Consumption and Healthy Lifestyles. Furthermore, the research is financially supported by the Dutch Cot Death Parent Association (VOWK).

## Conflict of Interest

The authors declare that the research was conducted in the absence of any commercial or financial relationships that could be construed as a potential conflict of interest.

## Publisher's Note

All claims expressed in this article are solely those of the authors and do not necessarily represent those of their affiliated organizations, or those of the publisher, the editors and the reviewers. Any product that may be evaluated in this article, or claim that may be made by its manufacturer, is not guaranteed or endorsed by the publisher.

## References

[1] Goldstein RD Blair PS Sens MA Shapiro-Mendoza CK Krous HF Rognum TO. Inconsistent classification of unexplained sudden deaths in infants and children hinders surveillance, prevention and research: recommendations from The 3rd International Congress on Sudden Infant and Child Death. Forensic Sci Med Pathol. (2019) 15:622–8. 10.1007/s12024-019-00156-9 31502215 PMC6872710

[2] Shapiro-Mendoza CK Palusci VJ Hoffman B Batra E Yester M Corey TS. Half century since SIDS: a reappraisal of terminology. Pediatrics. (2021) 148:e2021053746. 10.1542/peds.2021-053746 34544849 PMC8487943

[3] Taylor BJ Garstang J Engelberts A Obonai T Cote A Freemantle J. International comparison of sudden unexpected death in infancy rates using a newly proposed set of cause-of-death codes. Arch Dis Child. (2015) 100:1018–23. 10.1136/archdischild-2015-308239 26163119

[4] De Jonge GA Burgmeijer RJ Engelberts A Hoogenboezem J Kostense P Sprij A. Sleeping position for infants and cot death in The Netherlands 1985-91. Arch Dis Child. (1993) 69:660–3. 10.1136/adc.69.6.660 8285778 PMC1029649

[5] Central Bureau for Statistics Netherlands (2020). Deceased; Cause of Death (Comprehensive List) [Data set]. Available online at: https://opendata.cbs.nl/#/CBS/nl/dataset/7233/table

[6] Goldstein RD. (2018). Parental Grief. In: Duncan B. R. JR, editors. SIDS Sudden Infant and Early Childhood Death: The Past, the Present and the Future. Adelaide (AU): University of Adelaide Press. 10.20851/sids-08 30024688

[7] Horne RS. Sudden infant death syndrome: current perspectives. Int Med J. (2019) 49:433–8. 10.1111/imj.14248 30957377

[8] Ruys JH Engelberts AC van Velzen-Mol HWM. (2009). JGZ-richtlijn Preventie Wiegendood [YHC-guideline Prevention SIDS]. Gebaseerd op de gelijknamige richtlijn, opgesteld door de Nederlandse Vereniging voor Kindergeneeskunde en Artsen Jeugdgezondheidszorg Nederland in 2007. RIVM Rapport. 295001004.

[9] Veiligheid NL. (2019). De 4 van Veilig slapen. Kinderveiligheid. Available online at: https://www.veiligheid.nl/kinderveiligheid/slapen/veilig-slapen

[10] Wieske RC Nijnuis MG Carmiggelt BC Wagenaar-Fischer MM Boere-Boonekamp MM. Preventive youth health care in 11 European countries: an exploratory analysis. Int J Public Health. (2012) 57:637–41. 10.1007/s00038-011-0305-1 21956621 PMC3359454

[11] Blair PS Byard RW Fleming PJ. Sudden unexpected death in infancy (SUDI): suggested classification and applications to facilitate research activity. Forensic Sci Med Pathol. (2012) 8:312–5. 10.1007/s12024-011-9294-x 22076788

[12] Konijnendijk AA Engelberts AC L'Hoir MP Boere-Boonekamp MM. Elfde Peiling Veilig Slapen: waar en hoe leggen ouders hun kind te slapen? Nederlands Tijdschrift Voor Geneeskunde. (2018) 162:16–23. 30040267

[13] RIVM. (n.d.). volksgezondheidenzorg.info. Sociaaleconomische status. Available online at: https://www.volksgezondheidenzorg.info/onderwerp/sociaaleconomische-status/cijfers-context/opleiding#definities

[14] Central Bureau for Statistics Netherlands (2020). Population; Education Level [Data set]. Available online at: https://opendata.cbs.nl/statline/#/CBS/nl/dataset/82275NED/table?fromstatweb

[15] RIVM. (n.d.). volksgezondheidenzorg.info. Roken. Available online at: https://www.volksgezondheidenzorg.info/onderwerp/roken/cijfers-context/huidige-situatie-volwassenen#node-roken-naar-opleiding

[16] Moon R. Y. Task Force on Sudden Infant Death Syndrome. (2016). SIDS and other sleep-related infant deaths: evidence base for 2016 updated recommendations for a safe infant sleeping environment. Pediatrics. 138:e20162938. 10.1542/peds.2016-2940 27940805

[17] Alm B Wennergren G Möllborg P Lagercrantz H. Breastfeeding and dummy use have a protective effect on sudden infant death syndrome. Acta Paediatr. (2016) 105:31–8. 10.1111/apa.13124 26175065 PMC5049485

[18] Moon RY Horne RS Hauck FR. Sudden infant death syndrome. Lancet. (2007) 370:1578–87. 10.1016/S0140-6736(07)61662-6 17980736

[19] Blair PS Sidebotham P Evason-Coombe C Edmonds M Heckstall-Smith EM Fleming P. Hazardous cosleeping environments and risk factors amenable to change: case-control study of SIDS in south west England. BMJ. (2009) 339:b3666. 10.1136/bmj.b3666 19826174 PMC2762037

[20] Mitchell EA. SIDS: past, present and future. Acta Paediatr. (2009) 98:1712–9. 10.1111/j.1651-2227.2009.01503.x 19807704

[21] De Jonge G L'Hoir M Ruys J Semmekrot B. (2002). Wiegendood, ervaringen en inzichten. Den Haag: Stitching Wiegendood.

[22] Van Schaijk M Lanting C van Wouwe J Engelberts A L'Hoir M. Peiling risicofactoren wiegendood bij zuigelingen November 2002-april 2003. Leiden: TNO (2006). Available online at: http://resolver.tudelft.nl/uuid:2af22695-4f9c-49e7-be8e-8e1655796738

[23] Carpenter R McGarvey C Mitchell EA Tappin DM Vennemann MM Smuk M. Bed sharing when parents do not smoke: is there a risk of SIDS? An individual level analysis of five major case–control studies. BMJ Open. (2013) 3:e002299. 10.1136/bmjopen-2012-002299 23793691 PMC3657670

[24] Peeters D. Lanting C. Van Wouwe J. (2015). Peiling melkvoeding van zuigelingen 2015: Leiden: TNO.

[25] Tonkin S Gunn T Bennet L Vogel S Gunn A. A review of the anatomy of the upper airway in early infancy and its possible relevance to SIDS. Early Hum Dev. (2002) 66:107–21. 10.1016/S0378-3782(01)00242-0 11872315

[26] Lijowska AS Reed NW Chiodini BAM Thach BT. Sequential arousal and airway-defensive behavior of infants in asphyxial sleep environments. J Applied Physiol. (1997) 83:219–28. 10.1152/jappl.1997.83.1.219 9216967

[27] Richardson HL Walker AM Horne RS. Maternal smoking impairs arousal patterns in sleeping infants. Sleep. (2009) 32:515–21. 10.1093/sleep/32.4.515 19413145 PMC2663655

[28] Franco P Kato I Richardson HL Yang JS Montemitro E Horne RS. Arousal from sleep mechanisms in infants. Sleep Med. (2010) 11:603–14. 10.1016/j.sleep.2009.12.014 20630799

[29] Scheers N Rutherford GW Kemp JS. Where should infants sleep? A comparison of risk for suffocation of infants sleeping in cribs, adult beds, and other sleeping locations. Pediatrics. (2003) 112:883–9. 10.1542/peds.112.4.883 14523181

[30] Mitchell E Taylor B Ford R Stewart A Becroft D Thompson J. Dummies and the sudden infant death syndrome. Arch Dis Child. (1993) 68:501–4. 10.1136/adc.68.4.501 8503676 PMC1029275

[31] L'Hoir M. Engelberts A. Van Well G. Damste P. Idema N. Westers P.. (1999). Dummy use, thumb sucking, mouth breathing and cot death. Eur. J. Pediatr. 158, 896–901. 10.1007/s004310051237 10541944

[32] Limeira AB Aguiar CM de Lima Bezerra NS Câmara AC. Association between breastfeeding and the development of breathing patterns in children. Eur J Pedia. (2013) 172:519–24. 10.1007/s00431-012-1919-x 23274436

[33] McIntosh CG Tonkin SL Gunn AJ. What is the mechanism of sudden infant deaths associated with co-sleeping? The New Zealand Medical Journal (Online). (2009) 122:69–75. 20148046

[34] L'hoir M. Engelberts A. Van Well G. McClelland S. Westers P. Dandachli T.. (1998). Risk and preventive factors for cot death in The Netherlands, a low-incidence country. Eur. J. Pediatr. 157, 681–688. 10.1007/s004310050911 9727856

[35] Abed BZ Oneto S Abreu AR Chediak AD. How might non nutritional sucking protect from sudden infant death syndrome. Med Hypotheses. (2020) 143:109868. 10.1016/j.mehy.2020.109868 32480251

[36] Mcgarvey C McDonnell M Chong A O'Regan M Matthews T. Factors relating to the infant's last sleep environment in sudden infant death syndrome in the Republic of Ireland. Arch Dis Child. (2003) 88:1058–64. 10.1136/adc.88.12.1058 14670769 PMC1719406

