# Supplementary material for: Risk and Preventive Factors for SUDI: Need We Adjust the Current Prevention Advice in a Low-Incidence Country
Source: Front Pediatr. 2021 Nov 16;9:758048. doi: 10.3389/fped.2021.758048 (PMC8635138; doi:10.3389/fped.2021.758048)
Supplement: Supplementary file 1 [file Table_1.DOCX]

Supplementary Material

# Supplementary Figures and Tables

**Supplementary Table 1.** Prevalence in cases and controls of bed-sharing, stratified for age, birthweight, breastfeeding and smoking of mother; and the Odds Ratio (OR), both crude and adjusted in these strata.

|  | **Cases** | **Controls** | **OR** | **Adjusted OR^*^** |
| --- | --- | --- | --- | --- |
| Bed-sharing  *yes vs no* | 7 (16.3%) | 118 (10.0%) | 1.8 (0.8-4.0) | 2.0 (0.8-4.7) |
| *Age <4 mo* | 6 (26.1%) | 44 (9.1%) | 3.5 (1.3-9.4) | 3.3 (1.1-9.3) |
| *Age ≥4 mo* | 1 (5.0%) | 75 (10.6%) | 0.4 (0.1-3.4) | 0.5 (0.1-4.2) |
| *Birthweight <2500 gr* | 1 (11.1%) | 3 (6.2%) | 1.9 (0.2-20.4) | 1.4 (0.1-21.9) |
| *Birthweight ≥2500 gr* | 6 (17.6%) | 115 (10.2%) | 1.9 (0.8-4.7) | 2.0 (0.8-5.0) |
| *Any breastfeeding* | 1 (9.1%) | 98 (20.1%) | 0.4 (0.1-3.1) | 0.6 (0.1-4.8) |
| *No breastfeeding* | 6 (20.0%) | 20 (2.9%) | 8.4 (3.1-22.9) | 9.2 (3.0-28.6) |
| *Mother smokes* | 5 (35.7%) | 8 (10.7%) | 4.6 (1.2-17.1) | 17.7 (1.9-162.8) |
| *Mother doesn't smoke* | 2 (8.0%) | 110 (10.0%) | 0.8 (0.2-3.4) | 0.8 (0.2-3.4) |
| *Mother smoked during pregnancy* | 6 (40.0%) | 6 (13.5%) | 4.3 (1.1-16.3) | 10.8 (1.4-81.3) |
| *Mother didn’t smoke during pregnancy* | 1 (3.6%) | 112 (9.9%) | 0.4 (0.1-3.0) | 0.4 (0.1-3.2) |

* Adjusted for infant age, gender, birthweight and birth rank.
